# A Comparative Study of Visual Identification Methods for Highly Similar Engine Tubes in Aircraft Maintenance, Repair and Overhaul

**DOI:** 10.3390/s23156779

**Published:** 2023-07-28

**Authors:** Philipp Prünte, Daniel Schoepflin, Thorsten Schüppstuhl

**Affiliations:** 1 Institute of Aircraft Production Technology, Hamburg University of Technology, Denickestr. 17, 21073 Hamburg, Germany; daniel.schoepflin@tuhh.de (D.S.); schueppstuhl@tuhh.de (T.S.); 2 Lufthansa Technik AG, Weg beim Jäger 193, 22335 Hamburg, Germany

**Keywords:** visual part identification, similar object, object-inherent features, machine vision, neural networks

## Abstract

Unique identification of machine parts is critical to production and maintenance, repair and overhaul (MRO) processes in the aerospace industry. Despite recent advances in automating these identification processes, many are still performed manually. This is time-consuming, labour-intensive and prone to error, particularly when dealing with visually similar objects that lack distinctive features or markings or when dealing with parts that lack readable identifiers due to factors such as dirt, wear and discolouration. Automation of these processes has the potential to alleviate these problems. However, due to the high visual similarity of components in the aerospace industry, commonly used object identifiers are not directly transferable to this domain. This work focuses on the challenging component spectrum engine tubes and aims to understand which identification method using only object-inherent properties can be applied to such problems. Therefore, this work investigates and proposes a comprehensive set of methods using 2D image or 3D point cloud data, incorporating digital image processing and deep learning approaches. Each of these methods is implemented to address the identification problem. A comprehensive benchmark problem is presented, consisting of a set of visually similar demonstrator tubes, which lack distinctive visual features or markers and pose a challenge to the different methods. We evaluate the performance of each algorithm to determine its potential applicability to the target domain and problem statement. Our results indicate a clear superiority of 3D approaches over 2D image analysis approaches, with PointNet and point cloud alignment achieving the best results in the benchmark.

## 1. Introduction

For industrial applications, the unambiguous identification of parts is of great importance to allow efficient assembly and maintenance processes as well as traceability and task supervision. Typically, industrial components are identified through their unique serial or part number (P/N) that can, for example, be read from imprinted 1D/2D codes, Radio-Frequency Identification (RFID) tags or attached stickers that indicate the component type. A major challenge in part identification is that some components do not feature a part number, and identifiers get lost or are unreadable due to dirt, wear and discolouration. A possible solution is markerless identification using visual object-inherent characteristics [1,2]. The identification tasks can become quite demanding for objects that share a high degree of visual similarity if no distinct and unique features or markers are present.

This work is motivated by aircraft maintenance, repair and overhaul (MRO) processes, in which the major share of tasks is carried out manually and requires the expertise and knowledge of a skilled and experienced employee. Aircraft engine tubes inspected during MRO processes form an especially demanding target domain for identification as they share high visual similarity with each other and have multiple resting positions, movable flanges and a non-unique colour scheme. After disassembly, engine tubes must be cleaned before their inspection. The determination of the correct type is therefore required to choose the corresponding cleaning and inspection procedures. Currently, the identification task is performed manually through a visual check. During operation in an aircraft, P/Ns often become unreadable or damaged, so the identification becomes a time-consuming, personnel-intensive and error-prone task as component recognition without distinct features is difficult. There is huge potential for improvement through the application of modern identification techniques based on machine vision. Generally, identifying industrial components without additional markers allows for multiple tasks such as automated supervision of assembly tasks, quality control, part sorting [1,3] or robotic bin picking to be carried out.

Advances in markerless object recognition can be used to develop all kinds of applications in areas such as production, logistics, maintenance and quality assurance in a wide range of industries where the usage of markers is not feasible or inconvenient. Various image processing and machine learning techniques can be used to identify such objects in industrial processes. However, mainly due to the high visual similarity of components in the aircraft industry, commonly used object identifiers lack direct transferability towards this domain. With different approaches being successfully used for regular object identification in principle, it is unclear which of those are applicable despite such challenges. In the literature, there is very little information about the object recognition of highly similar industrial objects [2,4]. Moreover, scene context can also influence performance, yet there is only little available data dealing with industrial applications [5].

Therefore, this work presents an analysis and investigation of possible approaches for identifying visually highly similar components. This contribution aims to close the knowledge gap between the availability and applicability of possible recognition strategies for this difficult target domain.

For this purpose, the properties of aircraft engine pipes are analysed and characterised, and suitable approaches are implemented and then investigated concerning their potential suitability for a target domain characterised by a high degree of similarity and few distinct features. We aim to provide a proof-of-concept evaluation for different approaches solely based on object-inherent properties and provide a comparison using a small test dataset. We then assess and discuss the approaches’ difficulties to provide information on how to use those for later practical applications, potential improvements and in further research. With our contribution, we show that the proposed approaches are in principle suitable for identifying such components despite the lack of characteristic visual features. We also propose a human-in-the-loop system based on our findings suitable for practical application.

The rest of this paper is organised as follows: Section 2 introduces related work for object identification in an industrial context with a focus on settings that feature objects with a high degree of similarity among each other. Then, the identification concept and suitable approaches alongside the dataset used for evaluation are presented in Section 3. We explain and discuss possible approaches and methods to find those suitable for implementation and perform a comparison with our test data. Following this, Section 4 describes the implementation details and pipelines of the developed and investigated approaches. The results obtained with the proposed identification techniques using our 2D and 3D test datasets are presented in Section 5 alongside a discussion about possible improvements and further work required. Then, this contribution is closed with a conclusion in Section 6 that summarizes the most important findings, discusses practical applicability and presents a future outlook.

## 2. Related Work

For object recognition using 2D images, comparatively simple approaches such as image feature matching, template matching or bag-of-visual-words pipelines can be used. Another possibility is to use object contours and silhouettes seen from a bird’s eye view (BEV) or the calculation and comparison of geometric properties, such as the spatial extent. In the context of industrial applications, Ref. [6] proposes the use of simple geometric properties and contour outlines for the industrial sorting of regular geometric workpieces. The application of template matching for a part-sorting system is demonstrated in [7] using images from BEV perspective. The authors in [8,9] propose a framework for machine product inspection based on features computed from colours and textures. A prototype for object identification in logistics and warehouse management is presented in [1]. Their system uses multiple cameras and a scale to gather the objects’ physical dimensions and applies image feature matching for comparison with a pre-recorded database of known objects. The detection of unique, characteristic image keypoints and their description with algorithms such as Scale-Invariant Feature Transform (SIFT) [10] or Oriented FAST and Rotated BRIEF (ORB) [11] and a following comparison with reference images establishing correspondences is a common strategy often used for the processing of stereo images, object detection and tracking or panorama stitching. The concept is further extended by [3] with the introduction of a Convolutional Neural Network (CNN) for image processing.

Another commonly used method is direct image classification with neural networks such as CNNs. The rising popularity of Deep Convolutional Neural Networks (DCNN) led to an increase in openly available image classification architectures such as VGG [12], ResNet [13], MobileNet [14], DenseNet [15] and InceptionNet [16]. With their capability to handle complex scenes and a variety of use cases, their industrial applicability is sufficiently proven. A major drawback is the required extensive training and the availability of training data. In [17], this problem is addressed by using synthetic data. The successful use of synthetic training data for the domain of aircraft production and logistic scenarios is addressed by [18,19]. A variety of application scenarios for object identification of industrial components with a high degree of similarity using CNNs are shown in [2,4,20,21,22,23,24]. A very common application is the identification of fasteners and screws. In [4], the CNN-based identification of screws, washers and nuts using greyscale images is demonstrated. Another approach for screw-type classification in disassembly processes [23] employs top-view images, Hough-transform to obtain screw candidate proposals and a CNN for the final classification. A comparison of different CNN models for fastener and washer categorisation in aircraft overhaul processes is presented in [24]. Other applications include the visual identification of electric power fuses [20] and the classification of a variety of electro-mechanical components that feature high intra- and inter-class similarity [22].

Similar to [17], the work of [2] focuses on classifying small parts such as screws and nuts. Their novel contribution is the inclusion of a reference object in the identification process with a CNN. The resulting heavily improved classification accuracy indicates that this approach is promising. A related approach is employed in [21] that shows the classification of bolts and nuts with a CNN and also uses a reference object for visual calibration. The possible applicability of DCNN for identifying similar components can also be inferred from the works of [17], where small-scale automotive parts are identified through a multi-stage analysis pipeline. They compare different CNN architectures and identify challenging component properties such as (1) similar geometric scale, (2) being geometrically identical and only distinguishable by colour and (3) geometrically mirrored parts. Following these challenges, the work of [25] contributes a methodology to identify such identification challenges and suggests using multi-stage classification algorithms.

Sensor fusion of multiple perspectives and sensors is used in a system presented by [17]. As this work focuses on a comparative study between multiple methods for similar components, such a sophisticated and individually engineered multi-stage pipeline is out of the scope of this work. Nevertheless, the challenges identified in that work remain valid for the application domain that we address in this work. Further investigations regarding the applicability of deep-learning-based architectures to such use cases are needed. In addition, the emergence and promising results of transformer architectures may indicate a suitable alternative to the limitations that result from the use of DCNNs and will also be investigated in this study as existing studies focus on CNNs for the given task. Another alternative approach to the direct application of CNNs is the usage of siamese neural networks as proposed in [26]. Such a system uses two neural networks with the same weights to allow for the comparison of an image with a reference image by computing the distance between the feature vectors.

Regarding the usage of 3D data, a database designed for industrial object identification and pose estimation use cases is presented in [5] as there is a deficit of databases for industrial use cases and scene context is considered an important factor for successful object identification. The authors also apply shape-based 3D matching, point-pair voting and RANdom SAmple Consensus (RANSAC)-based feature matching as exemplary object recognition algorithms. An approach that detects geometric keypoints in point clouds and compares them with 3D models is introduced in [27]. A pipeline for 3D object recognition using only 2D camera images [28] combines scale-space and multiple virtual views from Computer-Aided Design (CAD) data for matching. The identification of similar mechanical parts in turbocharger CAD models using point clouds and a neural network is presented in [29]. The authors evaluate the applicability of PointNet to identify components in synthetically generated point clouds and show that it is suitable for such a task.

Based on the reviewed literature, it can be derived that the majority of the approaches and concepts are limited in certain parameters, rendering them unsuitable for the identification task at hand. Most of the approaches are constrained to images taken from BEV while investigating a target domain that has a limited spectrum of possible resting positions such as screws, washers and nuts [2,4,21,23,24]. As we have a multitude of previously unknown resting positions and quite complex pipe geometries, such approaches cannot be employed. Others, such as [8,9,22], rely on the presence of textures and colours—a feature not present within our target domain. Regarding the usage of 3D data for the classification of highly similar objects, there is a clear lack of studies in the literature. The evaluation in [29] shows that PointNet can be used for such a task, but the performance is assessed using synthetically generated point clouds; a real-world experiment with recorded data to prove applicability in practice is still missing.

## 3. Analysis, Concept Creation and Data Preparation

The investigated industrial use case that motivates this work deals with the identification of turbine pipes that need to undergo regular inspection, for which they are disassembled and removed from the aircraft. Following, all parts are roughly cleaned and pre-sorted to facilitate the subsequent identification and sorting required for cleaning and thorough inspection. Currently, a skilled employee carries out the identification task manually based on visual characteristics, markers and experience. To enable machine vision identification using either 2D or 3D data, the properties of the target domain need to be analysed to make a reasonable selection of suitable approaches. The following presents an analysis of the target domain, explains the fundamental ideas of potential identification methods and describes the selection made as well as the dataset used for evaluation.

### 3.1. Analysis of the Target Domain

**Figure 1:**
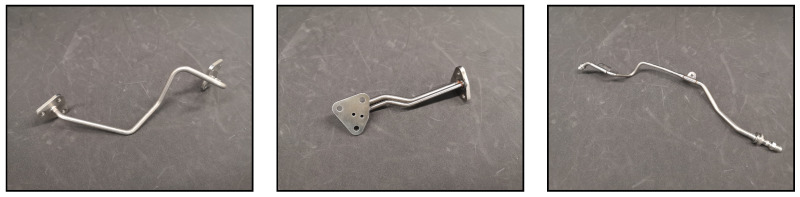
The target domain comprises aircraft engine pipes. They are characterized as long slender objects and differ with respect to their ends, drillings and attached mounting brackets and flanges. Discolouration, soling and wear of the textureless, metallic surface are common after flight operations.

The investigated target domain, exemplary images are displayed in Figure 1, consists of aircraft turbine pipes that can be described as long, slender objects with lengths ranging from a few centimetres up to about 80 cm. Most of the pipes have a much larger spatial extent in width and length as compared to their height. Nevertheless, some pipes also have significant extension in height and, therefore, a distinctively different appearance depending on the perspective chosen. Most of the pipes have two ends, but manifold pipes with multiple ends are also present. The pipe ends differ as there are threaded ends, fixed screw flanges, movable screw flanges, spigots, sockets and mandrels. The pipe surface is primarily metallic, highly reflective and without distinct textures. Discolouration, soiling and wear occur commonly but irregularly and, therefore, cannot serve as unique features. Due to an extension in all three spatial directions and the presence of flanges and mounting brackets, multiple resting positions are possible, as well as self-occlusion and casting of shadows depending on the lighting conditions.

Like in the current investigation and during the later practical application in MRO processes, often no CAD data or technical drawings are available, thus a set of demonstrator pipes is used for testing and evaluation purposes. First, a set of 15 2D demonstrator pipes bent from stainless steel tubes with diameters of 6 mm (6 parts) and 8 mm (9 parts) was available. All pipes had a metallic reflective surface and no further visible characteristics or modifications. In addition, another 3D demonstrator set was used, which was composed of ten 3D-printed pipes fabricated from black plastic with diameters of 8 mm, 12 mm and 15mm. For the sake of simplicity, no features such as flanges or threaded ends were added. The only visible characteristics were fine grooves caused by the individual printing layers.

### 3.2. Selection of Approaches

Various approaches to processing 2D or 3D data are conceivable for the object identification task. Relevant criteria for the selection are mainly the expected performance for the presented target domain and later applicability in MRO processes. In addition, the availability of required data, algorithms for data processing and the enabling effort are considered important factors. As the target domain is poorly studied, approaches offering good traceability and understandability capabilities, as well as possibilities for visualization, are preferred as they can provide useful information regarding their later applicability and possible application limitations. Visual object recognition approaches can be categorised into methods processing 2D data and algorithms designed for 3D data, among which suitable techniques must be selected.

#### 3.2.1. 2D Methods

As introduced in Section 2, comparatively simple approaches such as template matching, a comparison of calculated geometric features (e.g., spatial extent or covered area) or properties describing the contour or silhouette [6] using BEV images are conceivable for the 2D domain. In addition, the application of image feature matching [1,3] with algorithms such as SIFT [10] and ORB [11] can be used to recognize known objects by comparison with reference images establishing correspondences and evaluating the matching quality. For image feature matching, the availability of unique image characteristics within the images, as well as sufficient and realistic image captures, is essential.

In addition, AI-based solutions with CNNs [2,3,4,17] and transformer networks can be used. With the help of neural networks, image content can be directly processed and classified. However, this requires prior training with a sufficiently large dataset that describes the target domain as completely as possible. To address the problem of unavailable training data, synthetic generation is a possible solution [18,19].

#### 3.2.2. 3D Methods

Other promising approaches rely on processing 3D data, such as point clouds. In principle, two methods for the task can be distinguished. The first uses a comparison of acquired point clouds with reference point clouds establishing correspondences between the recording and reference. Similar to 2D image feature matching, keypoints can be used here to establish correspondences [27,30] or shape primitives found in the point clouds are feasible for similarity assessment [31]. An evaluation of the matching quality allows to determine whether the object of interest is contained within the recorded point cloud. The second approach for 3D data uses neural networks for classification. Exemplary networks are VoxNet [32], 3D ShapeNets [33], DeepShape [34] or PointNet [35] and its successor PointNet++ [36]. Here, the neural networks are trained with huge amounts of training data to allow direct classification of point clouds, given the training dataset sufficiently represents the target domain.

#### 3.2.3. Selection

A common problem with the 2D approaches is mediocre expectable performance. The target domain dealt with during MRO processes is composed of 3D objects with multiple resting positions, self-occlusions and strong differences in appearance based on the chosen recording perspective. Additionally, only very few characteristic, unique features or image keypoints that are needed for both the application of CNNs and image feature matching approaches can be found on the pipes. Discolouration, damage and a high visual resemblance to each other are major challenges for CNNs and image feature matching approaches. Nevertheless, classification employing geometric properties and pipe-specific features derived from the object contours and silhouettes seems promising due to its simple calculation and the comprehensibility of the decision making using such features. In addition, the application of a 2D transformer network seems promising since such networks are capable of dividing the image into multiple patches and thus can detect patterns despite multiple resting positions and views. As a simplification, only 2D demonstrator pipes will be investigated for this purpose to evaluate principle applicability.

As it can be expected that 3D approaches are best suited for the identification task at hand, two methods for processing 3D point clouds will be implemented and investigated in detail. The first is based on the comparison of point clouds with a database using Fast Point Feature Histogram (FPFH) keypoints. The FPFH feature descriptor [30] is a popular and widely used 3D keypoint detection algorithm for which an implementation is available in Open3D [37]. Matching and comparison with a reference can then be carried out through point cloud registration and alignment and will be described in more detail in the following section. The second promising option to be investigated is classification with a neural network. PointNet [35] is chosen as an exemplary network as it is widely used in various domains, is well documented and is also used for similar industrial object identification tasks [29].

### 3.3. Dataset

Over the course of this paper, two recorded and an additional synthetic dataset are used. The first dataset contains 2D images and the second 3D point clouds. In the following, these are referred to as 2D dataset and 3D dataset, respectively. For both datasets, a class-balanced portion of 70% of the data is used as the train-split, and the remaining 30% serves as the test-split. The synthetic 2D dataset contains additional computer-generated 2D images to enable the training of data-driven approaches. For all datasets, we apply extensive data augmentation described in more detail for each of the data-driven approaches to allow the models to be capable of generalizing during training.

**Figure 2:**
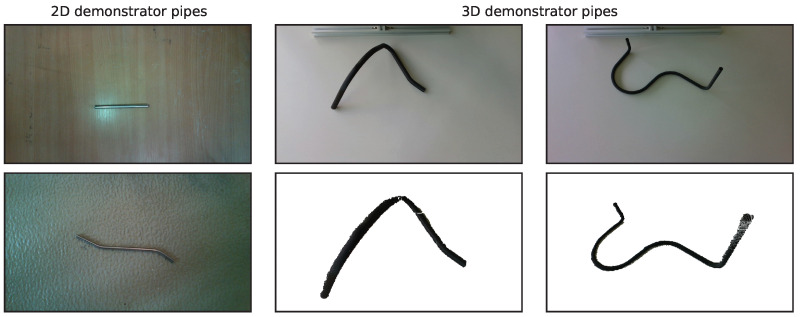
Exemplary images for both datasets. Left column: two images taken from the 2D dataset containing 2D demonstrator pipes. Right two columns: two images and the respective extracted and filtered point clouds of two pipes contained in the 3D dataset.

The 2D dataset is composed of BEV images of the 2D demonstrator pipes taken from a fixed distance. Each pipe is assigned a class number ranging from 1 to 15. The pipes are placed on different backgrounds that can be found in a workshop, namely a wooden table surface, a white laboratory table surface and a workshop floor. For each pipe and background, 10 images are taken, except for classes 1 and 12, as these lack diversity due to their simple geometry (straight tubes), so only 8 images each are taken. Exemplary images can be found in Figure 2. It should be noted that the omission of some class 1 and 12 recordings leads to a slight class imbalance, which should generally be avoided for data-driven approaches. However, the data quality should be sufficient for the intended demonstration of potentially applicable methods and a subsequent discussion.

The 3D dataset is composed of point clouds of each 3D demonstrator pipe, designated as classes 1 to 10. For each class, 20 recordings are obtained, resulting in a total of 200 point clouds. The demonstrator pipes are digitized with an Intel D435i stereo camera mounted on a frame in an oblique downward perspective while the pipes are lying on a white workshop table. Between each recording, the pipes are moved, rotated and repositioned by hand. A simple colour threshold segmentation algorithm is applied to extract the points belonging to the pipes to obtain the final point clouds. Exemplary recordings and extracted point clouds can be seen in Figure 2.

## 4. Classification Pipelines and Implementations

As indicated in the previous section, in total four methods will be implemented—two methods for 2D images and two methods capable of processing point clouds. Besides different working principles, the type of reference data required for identification differs in particular. The 2D approaches and the PointNet neural network require realistic images or point clouds, respectively. The method based on point cloud alignment, on the other hand, depends on high-quality CAD data to be available.

### 4.1. Classification Using Geometry and Shape

The first approach is based on the description of the geometric properties of the pipe silhouettes seen from BEV using random forests for classification. In addition to generic features such as silhouette width, height, area or Hu-moments [38], domain-specific features such as pipe length or diameter are taken into account. As in the literature, no hand-crafted, domain-specific features for the target domain are presented; we derive such and add some of them to our classification pipeline. We then assess whether those features provide substantial benefits compared to automatic classification with generic geometric properties. A major advantage of using random forests (RF) over a single decision tree is that combining multiple predictions leads to a more reliable prediction since feature outliers can be better filtered out. In addition, a probability score is computed and used to assess the trustworthiness of predicted results.

#### 4.1.1. Domain-Specific Features

**Figure 3:**
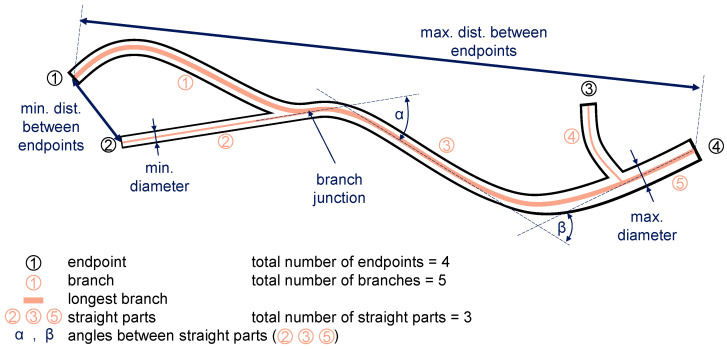
Illustration of domain-specific features. The lines in salmon colour represent the skeleton that describes the pipes’ main geometry.

The pipes in the target domain can be described as deformed and bent straight tubes with attachments, such as flanges or mounting brackets. A simpler representation can be given in the form of a skeleton that describes its main geometry. Figure 3 illustrates domain-specific features derived from the target domain, which are as follows:

- **Length of longest branch:** longest possible branch in the skeleton representation that connects two arbitrary pipe ends;
- **Minimum and maximum distance between two ends:** largest and smallest Euclidean distance between any of the pipe ends;
- **Number of branches:** number of pipe sections for multi-tube pipes or those having flanges and connectors that appear as branches in the computed skeleton;
- **Number of ends:** number of pipe ends for multi-tube pipes;
- **Number of straight parts:** number of straight pipe sections;
- **Angles:** the angles at which the straight sections are angled to each other;
- **Minimum, maximum and average pipe diameter**;
- **Histograms of branch lengths and pipe diameters**.

We propose to use skeletonization to determine the aforementioned domain-specific features as a skeleton’s simple structure allows us to obtain them easily. For example, diameters can be computed by determining the distance between the pipe’s main axis (the skeleton) and its silhouette contour. Since the 2D demonstrator pipes are a highly simplified representation of possibly much more complex pipes handled in MRO processes, only the longest branch length, the maximum distance between two ends and the average diameter are considered in the following for assessing the potential use of domain-specific features.

#### 4.1.2. Pipeline

**Figure 4:**
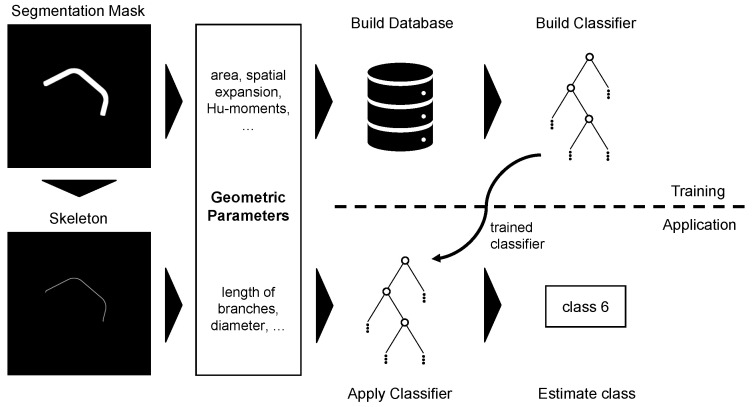
The pipeline of the geometric approach. The input is a binary segmentation mask from which the geometric properties are directly calculated or obtained with the help of a skeletonization algorithm.

The pipeline for the geometric approach depicted in Figure 4 uses a random forest (RF) classifier composed of 100 decision trees. The geometric features are computed from either the pipe silhouettes (generic features) or the skeleton (domain-specific features), which is obtained with the skeletonization algorithm from [39] implemented in scikit-image [40]. The recorded images are segmented using transfer learning with a modified UNet to obtain the silhouettes, as explained in [41]. In principle, other segmentation methods, such as chroma keying when using a monochrome background or contour detection with the Hough-transform or Canny-edge algorithms, are also conceivable for segmentation.

To enable this approach, the random forests are trained using the 2D dataset train-split. A possible data imbalance could be compensated for by applying class weights, but this was not applied here due to the only weak imbalance present. First, geometric features are computed and saved in a database, and then the database is used to train the RF’s weights. After training, new samples can be classified by computing their geometric features and processing them with the trained RF classifier. To assess the usability and significance of the domain-specific features, the RF classifier is built for three feature sets containing either (1) all geometric features, (2) only generic features or (3) only domain-specific features.

### 4.2. Classification Using Transformer Networks

As revised in Section 2, DCNNs struggle to distinguish very similar objects. Recently introduced transformer architectures are rising in popularity for vision applications. While DCNNs extract features using convolutional layers from locally confined image regions, vision transformers can employ attention between all image regions. With this global attention mechanism, they can capture inter-regional dependencies and more complex image features. With this capability to handle complex interactions between image regions, they may be able to distinguish similar components.

The implementation of this work follows [42] in the Hugging Face (HF) framework [43]. The HF framework is a high-level API for PyTorch that provides a wide range of pre-trained models and a simple interface for training and fine-tuning them. The HF framework is used to load a pre-trained Vision Transformer (ViT) model [42] and then train it on the 2D dataset. The image is split into patches, which are fed into a transformer encoder consisting of a multi-head self-attention layer and a feed-forward layer. The multi-head self-attention layer computes the attention between all patches, and the feed-forward layer computes an output for the transformer layer. The ViT model is pre-trained on the ImageNet dataset [44] and fine-tuned on the 2D dataset.

Due to heavy limitations regarding dataset size and class imbalance in the training dataset, data augmentation was used with the following augmentations:

- **RandomResizedCrop:** randomly crops the image to a given size and aspect ratio;
- **RandomHorizontalFlip:** randomly flips the image horizontally;
- **Normalize:** normalizes the image with the given mean and standard deviation;
- **ColorJitter:** randomly changes the brightness, contrast, saturation and hue of the image;
- **RandomRotation:** randomly rotates the image by a given angle.

A hyperparameter study was performed to find suitable hyperparameters for the ViT model:

1. Training batch size;
2. Learning rate;
3. Training epochs.

After the hyperparameter study, the best-performing hyperparameters were used to train the final ViT model on the 2D dataset. The ViT model was then evaluated on the 2D dataset test-split.

### 4.3. Classification Based on Point Cloud Alignment

**Figure 5:**
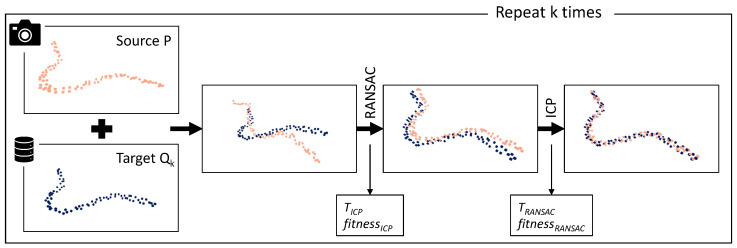
The pipeline using point cloud alignment for object recognition. The recorded point cloud *P* is aligned with every point cloud $Q_{k}$ saved in the database. First, a global alignment is computed. Following this, the alignment is refined, and the matching metrics needed for classification are computed.

The fundamental idea of this approach is to use point cloud alignment techniques originating from point cloud registration to find the best fit of a recorded point cloud *P* with any of the *k* reference point clouds $Q_{k}$ saved in a database. The applied alignment pipeline shown in Figure 5 and its initial parameters are based on the proposed pipeline for point cloud registration [45] of the Open3D library. It is adjusted and extended to fit the identification problem at hand. The database is built with CAD models from which point clouds are computed using the Poisson disk sampling technique implemented in Open3D.

In the first step, the recorded data are pre-processed. The recorded point cloud *P* is downsampled to obtain $P_{down}$, and the FPFH feature vectors are computed for each point in $P_{down}$. The downsampled point cloud and its feature vectors are then used to obtain a transformation matrix *T* that gives a coarse alignment with each of the *k* downsampled reference point clouds $Q_{k,down}$. The alignment uses the RANSAC algorithm, configured to pick three random feature points from $P_{down}$ to align them with the reference point cloud $Q_{k,down}$. RANSAC returns a transformation matrix *T*, and a set $C=\left\{(p,q)\right\}$ of corresponding point pairs where $p\in P_{down}$ and $q\in Q_{down}$. The alignment quality is described with the alignment metrics *fitness* and *inlier_rmse* as defined in Equation (1). The algorithm computes multiple transformation matrices *T* for each $\left(P_{down},Q_{k,down}\right)$ pair and the best fit in terms of *fitness* is returned.

(1) $$fitness=\frac{N_{c}}{N_{p}}\;\;inlier\_rmse=\sqrt{\frac{\sum_{(p,q)\in C}d_{p-q}^{2}}{N_{c}}}$$

In Equation (1), $N_{c}$ is the number of inlier correspondences, and $N_{p}$ is the number of points in $P_{down}$ (RANSAC) or *P* (ICP), respectively. For RANSAC, $p\in P_{down}$ and $q\in Q_{down}$, whereas ICP is applied for the full points clouds, so p∈P and q∈Q.

The coarse alignment obtained with RANSAC is then iteratively refined using Iterative Closest Point (ICP). The working procedure is as follows:

1. Find a correspondence set $C=\left\{(p,q)\right\}$ of corresponding point pairs where p∈P and q∈Q which have a distance smaller than a defined threshold.
2. Update the transformation *T* by minimizing an objective function. In this case, point-to-point ICP algorithm with objective function $E\left(T\right)=\sum_{(p,q)\in C}{\left|p-Tq\right|}^{2}$ is used.

After reaching a defined number of iterations or having too few relative improvements in terms of *fitness* or *inlier_rmse*, the algorithm terminates and returns the final alignment transformation matrix *T*. Once it has obtained the alignments with each reference model $Q_{k}$ and the corresponding *fitness* values, the object type is determined by utilizing the highest *fitness* value among all alignments.

To enable the proposed pipeline, an extensive parameter grid search for the parameters needed for downsampling, RANSAC and ICP is carried out using the dataset train-split.

### 4.4. Classification with PointNet

The next approach for part identification uses PointNet [35], a neural network designed for the direct classification and semantic segmentation of point clouds. The implementation is based on [46], and adjustments are made to allow hyperparameter optimization and compatibility with the available dataset. PointNet consumes raw point clouds, each composed of a fixed number of points. The network internally computes a global feature vector and outputs probability values for each class. A dense layer with a softmax activation function is then used to determine the estimated class. To enable this approach, the neural network needs to be trained on the target domain, and a set of suitable hyperparameters has to be found. Since the network consumes point clouds representing the pipes only seen from the current recording perspective, the network training shall contain as many different views during training as possible.

For training and during hyperparameter optimization, the dataset train-split is used and further divided into a set containing 11 point clouds per class used for training and a validation set with 3 point clouds per class used to find the best set of hyperparameters. As the amount of training and validation is limited due to few recordings per class, extensive data augmentation is applied to both sets to improve the network generalization capabilities and to extend the amount of available training and validation data. For each point cloud in the dataset, multiple point clouds are created by randomly drawing a set of points. Further augmentation is applied through random rotation, adding Gaussian noise and shuffling the point order.

In the first step, the optimal model hyperparameters are found using a grid search. The parameters are as follows:

- **Number of points per point cloud** *n*: determines the number of points drawn from the database point clouds as it is unknown how many points are required to allow the network to perform successful classification. The network input size is, therefore, equal to *n*.
- **Training batch size** *b*: the training batch size is a trade-off between the extent of weight variation and convergence during training.
- **Data augmentation factor** *f*: the amount of data augmentation refers to the number of point clouds created from each recorded point cloud in the dataset. It is desirable to have a large set of training samples, but the larger the training set, the longer the network will take to train.

After the grid search, the network is trained with the found hyperparameters and the best epoch is determined. In the last step, the dataset test-split is applied to verify the performance and assess suitability for the target domain.

## 5. Results and Discussion

In the following, the approaches presented in the previous Section 4 will be examined for their performance. For this purpose, the classifiers are built, trained and then checked with the dataset test-split.

### 5.1. Classification Using Geometry and Shape

The application of the test data from the 2D dataset yields classification accuracies of up to 99.2%, as presented in the first row of Table 1. That score can be achieved using all (generic and domain-specific) features as well as using only generic features. It can be deduced that, unlike anticipated, the additional domain-specific features do not contribute the significant difference needed for classification. Nevertheless, using only domain-specific features (in total, three parameters) still yields an accuracy of 80.9%, but this is far below the aforementioned scores.

**Figure 6:**
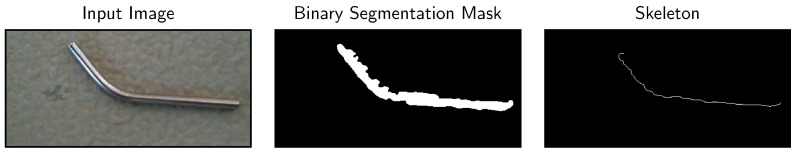
Misclassified class 2 pipe from 2D dataset due to an erroneous skeleton. The binary segmentation mask obtained has jagged edges and leads to an unsmooth skeleton that has a path length much larger than its nominal value.

**Figure 7:**
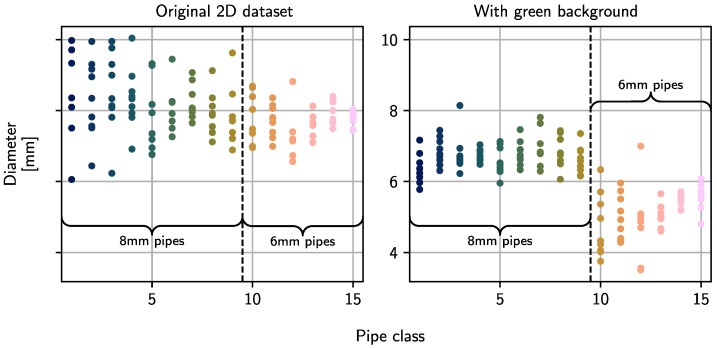
Computed diameters for 2D dataset and for an additional dataset recorded in front of a green background to apply chroma keying segmentation. Each dot represents an individual measurement. For the 2D dataset, no distinct diameter differences are visible. When using a green background, 6 mm and 8 mm pipes are clearly distinguishable, but the diameters are underestimated.

The reason for the insufficient performance using domain-specific features can be found in their calculation and the strong fluctuations in their values. The skeletonization algorithm consumes segmented images and thus essentially depends on the segmentation quality. Frayed and rough edges or fragmented pipe sections lead to distorted skeletons and erroneous feature values. Figure 6 shows that effect exemplarily, where the frayed segmentation mask leads to a jagged skeleton and, therefore, to a pipe length much larger than the nominal value. As an additional example, the calculated pipe diameters for multiple pipes from all classes are plotted in Figure 7. It can be observed that no distinct difference between 6 mm and 8 mm pipes is visible. To prove that the calculation of domain-specific features using a skeletonization approach works in principle and can be used for classification purposes, an additional dataset with the pipes being placed on a green background is recorded. Using a monochrome background and chroma keying segmentation leads to much smoother and cleaner segmentation masks. The classification results are given in the lower row in Table 1. Even though the accuracies are lower for the first two feature sets, a significant improvement can be achieved when using only domain-specific features. The estimated diameters for that dataset are also plotted in Figure 7. Now, a clear difference between the two diameter types is visible. A similar observation can be made for the other domain-specific features: branch length and distance between endpoints. Still, using only generic features yields better results, but, in principle, a classification using only the three selected custom-made features is feasible.

As an interim conclusion, it can be stated that the classification based on geometry and shape is possible in principle. Concerning the applicability to real pipes in MRO processes, however, it should be mentioned at this point that strong simplifications were made by using 2D images and 2D demonstrators. The possible poses of 3D components and the resulting pose variety as well as possible self-occlusions and also the restriction to only BEV recordings should be considered with regard to a possible application. Nevertheless, it is shown that domain-specific features can be useful since only three feature types were sufficient to achieve good classification results. It would also be conceivable to extend the pipe-specific parameters to 3D input data, such as point clouds, and compute properties, such as diameter, volume or length, in a similar manner using skeletonization.

### 5.2. Classification Using Transformer Networks

First, a hyperparameter search was conducted to find the best configuration for the ViT Transformer. The varied hyperparameters are provided in Section 4.2. The transformer was trained on an Nvidia A6000 GPU, with the HF framework [43]. The hyperparameter search was carried out using the Optuna Hyperparameter Framework [47]. The hyperparameter values that were investigated in the search are shown in Table 2, with model 24 resulting in the best accuracy.

#### 5.2.1. Training and Results of Final Model

The final model was trained for 250 epochs using the hyperparameters from the hyperparameter search. The training time was 30 min. The accuracies for training and testing on the test dataset are shown in Table 3.

**Figure 8:**
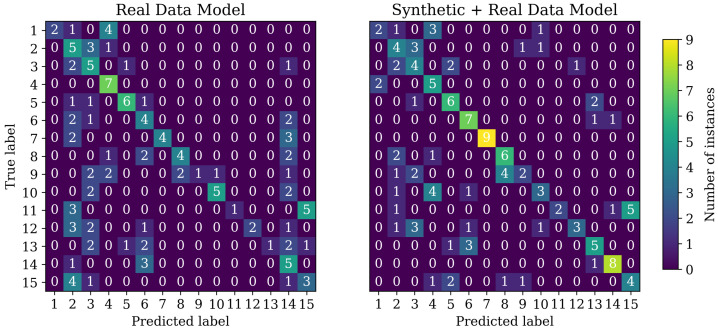
Confusion matrix of the ViT Transformer.

Application of the trained model on the test data yields the classification results shown in Table 3. The $acc_{test}=0.42$ performance is noticeably worse than the $acc_{val}=0.53$ performance. Since the accuracy is lower than those from other classification approaches, a more detailed analysis of the results was conducted. For this, the confusion matrix was used, which is shown in Figure 8.

The confusion matrix reveals that the model is not able to classify several classes of the problem reliably. Regarding misclassifications, it can be seen that the model frequently suggests classes 2, 3, 4, 6, 14 and 15.

#### 5.2.2. Improving ViT Transformer Results with Additional Synthetic Training Data

Due to the poor results of the ViT Transformer, additional synthetic training data were generated to test whether the poor performance was due to the few training samples. Synthetic data were generated using a previously reported toolbox from [18]. An additional 1000 samples were generated for each class. The training was carried out in two stages. First, the model was trained on the synthetic data with the same hyperparameters as in the previous training, as shown in Table 2. Afterwards, the model was fine-tuned on the real data. The results are shown in Table 3 in the row labelled *synthetic + real*.

The results show that additional synthetic data improve the performance of the model. The $acc_{val}$ increases from 0.53 to 0.62, and $acc_{test}$ improves from 0.42 to 0.53. The confusion matrix in Figure 8 shows that the model is now better at differentiating other classes from classes 2, 3, 4 and 6 with fewer false positives towards those classes. The performance for the classification of classes 7–13 is comparable to that of the real data model. Class 14 was significantly less predicted compared to the previous model and greatly increased the overall classification accuracy. Class 15 has the same misclassifications regarding class 10 as before.

#### 5.2.3. Discussion of the ViT Transformer Model’s Performance

Since the accuracies of the vision transformer result in rates of 42% and 53%, no successful use of the model in the addressed component classification use case could be established. The initial assumption, a lack of training data being the cause of the performance, and the attempt to mitigate this through synthetic data generation do improve the results, but not to a great extent. The results of the synthetic data model are still not sufficient for the industrial applicability of this use case. It is doubtful that further synthetic or real data would improve the results to a vastly different level. An end-to-end classification approach is thus not considered feasible.

However, since the network, in general, was able to learn how to differentiate between certain classes and the results are not completely random, applicability to less demanding use cases might be possible.

### 5.3. Classification Based on Point Cloud Alignment

#### 5.3.1. Hyperparameters

As many parameters influence the alignment procedure, a hyperparameter study using the dataset train-split was carried out to find the hyperparameters suitable for the dataset at hand. An important parameter is the number of points $n_{CAD}$ that are sampled from the CAD reference models to obtain point clouds used for computing the keypoints and alignment. Another parameter of interest is the number of iterations $i_{ransac}$ and $i_{icp}$ needed for proper alignment. Other parameters describe the FPFH neighbourhood size and distance thresholds applied to point correspondences. Since those are very specific to the dataset at hand, they are omitted in the following. An overview of the aforementioned parameters and their values for the hyperparameter search can be found in Table 4.

An important observation is that it is beneficial to have a sparsely sampled point cloud as a reference. It is not necessary to have thousands of points to find sufficient correspondences for alignment. A second finding is that a high number of iterations leads to higher-quality alignments with more reliable metrics and, consequently, also to successful class estimates. Further work should be carried out regarding lower and upper limits for that parameter, as it strongly determines the computational cost and time.

#### 5.3.2. Global vs. Local Alignment

**Figure 9:**
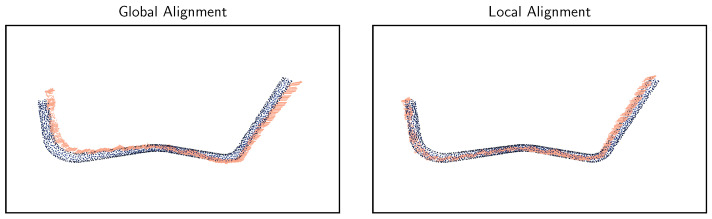
Successful alignment of a class 8 point cloud (salmon colour) with its reference model (blue).

Another interesting result is the comparison of the class estimation using the *fitness* metric from global registration with the RANSAC algorithm versus the subsequent refinement using ICP. The classification scores for five runs are given in Table 5. It can be observed that local refinement improves the accuracy by a huge margin and is therefore definitively recommended. The successful alignment of an exemplary point cloud can be seen in Figure 9.

Since it is especially important to avoid false positives, a threshold can be used to categorise predictions as invalid in case of low certainty, meaning relatively low *fitness* values. Based on results with the dataset train-split during the hyperparameter study, a value of 0.85 has proven to be suitable, as can be seen in Table 5.

#### 5.3.3. Application of Test Data

To evaluate this approach, a threshold value of 0.85 was applied to minimize false positives. Again, each point cloud in the test-split was classified five times to reduce the effect of randomness occurring during the application of RANSAC. The true-positive rate is 0.893, while the false-positive rate is 0.033. The remainder was categorised as invalid. The first important finding is that the proposed approach is suitable for the target domain and can provide a relatively reliable classification despite the absence of characteristics. Furthermore, it can be shown that CAD data can be used as reference data.

**Figure 10:**
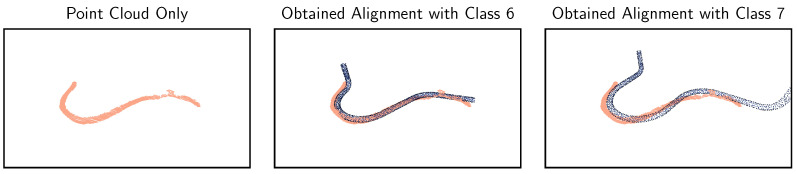
Alignments of a recorded class 6 point cloud (salmon colour) with CAD reference models (blue). In the upper left corner, portions of the pipe are missing due to inferior recording quality. For both alignments, *fitness* scores of 1 are obtained, as the recorded point clouds align tightly with both reference models so that all points are considered as inliers. The classification is therefore categorised as invalid.

Visualizing the false positives and invalid estimates, it turns out that the errors can be clustered into some groups and are mainly caused due to inferior data quality. A common issue is missing portions of the pipes, leading to erroneous alignments. Another consequence is that the pipes look too similar in some cases because characteristic pipe sections were recorded incorrectly, as visualized in Figure 10. Partial geometry overlap of the pipes is a general challenge that could be addressed by tightening the alignment hyperparameters. On the other hand, that would require better recording quality with continuous and smooth surfaces. Nevertheless, it is shown that despite the partially low recording quality and faulty recordings, the approach is, in principle, suitable for the classification problem at hand.

### 5.4. Classification with PointNet

#### 5.4.1. Hyperparameter Optimization

The network was trained from scratch with each of the 18 parameter combinations using grid search given the values in Table 6. To allow a better comparison of the validation accuracies obtained in the last epoch, smoothing with an exponential moving average and a factor of 0.85 was applied. The computing infrastructure uses a Nvidia GeForce RTX 3090 graphics card and Tensorflow 2.6.

The best validation accuracy $acc_{val,17}=0.954$ is obtained for model 17 with point clouds of size n=2048, training batch size b=128 and as much data augmentation as possible (f=100). Comparing the models’ validation accuracies, it can be observed that larger point clouds and extensive data augmentation lead to significantly better performance. The six worst models are those with very little data augmentation (f=10), while the performance increases for larger *n* and *f*. Interestingly, the third best network with much smaller n=128, b=32 and f=100 still achieves $acc_{val,2}=0.880$ while taking only about a quarter of the training time of model 17. Even though the performance of model 2 is clearly lower, it is worth considering both models for training as the smaller, lightweight model might also achieve an acceptable performance given sufficient training while offering a largely reduced training time.

#### 5.4.2. Training of Selected Models

Since it was found during the hyperparameter search that extensive data augmentation leads to significant performance improvements, the extent of data augmentation was further increased and set to f=500 for training both models. After 800 epochs of training, the best epoch was selected using the best validation accuracy $acc_{val}$. The results for training and validation accuracies, $acc_{train}$ and $acc_{val}$, are given in Table 7.

#### 5.4.3. Application of Test Data

The application of the test-split using the best epochs for each model reveals an unexpectedly high difference between the validation accuracy $acc_{val}$ and test accuracy $acc_{test}$ for both models; values are given in Table 7. The only difference between the validation and test data is the data augmentation procedure applied only to the training and validation data. Therefore, the reason must be found in the augmentation process that apparently modifies the data too excessively. Since PointNet is designed to be point-order- and pose-invariant, the added noise can be determined as the reason for the observed behaviour. Adding the same amount of noise to the test data, the obtained accuracy $acc_{test,noise}$ increases by a huge margin, as displayed in the right columns in Table 7. Initially, the noise was thought to be a useful data augmentation technique, but it is shown here that the training data differ too much from the application data. This is not necessarily a disadvantage, as noise can be imagined as adding some variation during training, but it needs to be considered for application as well. A second interesting observation is that the initial assumption that the small model 2 might be capable of achieving satisfactory performance is proven wrong. The small network with n=128 points performs significantly worse than the larger model 17. In the original paper about PointNet [35], the authors explain that there exists a lower and an upper bound needed to yield the same global feature vector which is used for classification. If the point cloud is sampled too sparsely, as seems to be the case here, critical points are lost and the global feature vector changes. Further investigation regarding this issue should be conducted in a separate study.

Taking a closer look at the misclassified point clouds with noise applied reveals that the PointNet approach also seems to have problems with recordings that are split into multiple point clusters due to inferior recording quality or missing portions of the pipes. Nevertheless, for the larger model 17, only two point clouds were misclassified, meaning that PointNet is capable of generalizing quite well, resulting in a better recognition performance than the previous approach. In trying to identify and cluster the problems, model 2 also suffers from recorded point clouds of poor recording quality and missing portions of the pipe. Nevertheless, there are also point clouds for which no specific pattern can be identified, so it is derived that the issues arise from the smaller input dimension and the resulting information loss. It should be noted at this point that the classification using PointNet performs better and is faster during application than using point cloud alignment, but requires extensive training and computation power.

## 6. Conclusions

In the course of this work, different approaches for the visual recognition and classification of highly similar parts have been investigated and tested using a set of simplified demonstrator pipes. It is shown that the classification task is possible even without distinct, characteristic visual features using modern computer vision and machine learning techniques. While the most simple approach based on hand-crafted geometry and shape features computed from 2D images works well for simple demonstrator pipes, it is deduced that this technique is not directly applicable to real-world parts due to the expected poor performance and lack of sufficient features resulting from the diverse resting positions and possible occlusions. The experiments conducted with transformer networks suggest that this approach has problems with the unambiguous recognition of the investigated domain. In addition, pre-training with synthetic data does not notably boost the network’s performance. Therefore, a practical application does not seem to be possible at the moment.

On the other hand, 3D approaches show great potential in classifying highly similar components, with successful applications using both PointNet and point cloud alignment approaches. The high recognition performance for the 3D demonstrator pipes having very few distinct features shows that no prominent, visible characteristics are necessary. While the performance using PointNet is slightly better, the point cloud alignment approach has some individual advantages regarding the avoidance of false positives, good visualization capabilities and simple extensibility of the classifier with additional CAD models, as no training is required. The neural network, on the other hand, is very fast in processing the point cloud to be analysed and can be trained with recorded point clouds, meaning that no CAD models are necessary, but it requires a lengthy training phase. Serious disadvantages, however, are the inadequate detection of incorrectly classified parts and the difficulty of adding new components.

**Figure 11:**
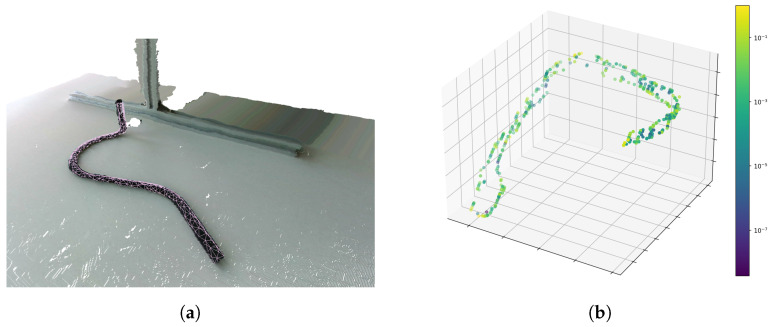
(**a**) Possible enhancement using an alignment visualization with a CAD model mesh to visualize the obtained alignment and allow the user to quickly assess whether the classification can be trusted. (**b**) Future research activity regarding feature importance. Application of an integrated gradients method to compute the contribution of each point to the classification result. The colours visualize importance, while larger values indicate a higher contribution.

A combination of both approaches seems promising for a human-in-the-loop system that can assist a human with the classification task and reduce the number of guesses, comparisons and cross-checks needed to determine the correct class. In such a system, PointNet can be used for the initial classification, while the point cloud alignment approach can then be employed to visualize the obtained result as illustrated in Figure 11a. Visualizing the aligned CAD model with the recorded point cloud allows a human to verify very quickly if the two parts are identical. The user then reviews and flags the visualization to avoid misclassifications. In the long term, this also helps to record more flagged data for further training and thus reduce required manual interaction in the future. However, there is still a need for further research regarding the use of synthetic point cloud training data based on CAD models and the reduction of false positives. In addition, research using a bigger dataset and a study with recordings from real-world use-case-related pipes would be beneficial to prove applicability in practice. Another interesting research area is the explainability of neural networks, especially for highly similar components. Methods such as SHapley Additive exPlanations (SHAP) [48] or Integrated Gradients [49], for example, could be used for conducting such studies and help to gain a deeper understanding of the decision making in neural networks for use cases with visually similar components. To illustrate that point, the per-point importance of an exemplary recording is visualized in Figure 11b. It can be observed that especially the pipe ends and the buckling in the rear have high importance values and therefore contribute significantly to the final class estimate.

Possible enhancements, in general, include the improvement of image and point cloud segmentation and the extension of the geometric approach to 3D data. The usage of a pre-sorting stage based on simple geometric features also seems feasible and allows for complexity to be reduced in later stages. In addition, visualization with CAD reference data, as illustrated in Figure 11a, could be beneficial for later practical application as the applicant can quickly assess if the reference and recording visually match. The improvement of the point cloud recording quality, as well as the early filtering of low-quality recordings, can also help to boost the performance of a later practical application. A clustering algorithm can help to determine if the recorded point cloud is separated into multiple clusters and is therefore missing significant portions relevant for classification.

## Figures and Tables

**Table 1:** Classification scores for test data using geometry and shape-based approach.

Description	All Features	Only Generic Features	Only Domain-Specific Features
2D dataset test-split	0.992	0.992	0.809
Chroma keying with green background	0.963	0.963	0.948

**Table 2:** Results of the hyperparameter search for the ViT Transformer.

Parameter Name	Values	Best Value (Trial 24)
number trials	30	-
batch size	[8, 16, 32, 64, 128]	32
learning rate	[0.000005, 0.0001]	0.00009688
epochs	[30, 50, 100, 250]	250

**Table 3:** Accuracies of the final ViT model training after 250 epochs.

Model	Train Time	${\mathrm{acc}}_{\mathrm{val}}$	${\mathrm{acc}}_{\mathrm{test}}$
24	30 min	0.53	0.42
synthetic + real	90 min	0.62	0.53

**Table 4:** Investigated hyperparameters for the point cloud alignment approach.

Parameter	Values	Best Value
$n_{CAD}$	[100, 5000, 10,000]	100
$i_{ransac}$	[1000, 50,000]	50,000
$i_{icp}$	[10, 30, 100]	100

**Table 5:** Classification scores on dataset train-split using the best hyperparameter combination and *fitness* alignment metric. Each component is classified three times for the global score and five times for the local score to reduce the effects of randomness. In addition, threshold values are applied to categorise uncertain estimates as invalid and avoid false positives.

Result	Global	Local	Local and Thresh. 0.85	Local and Thresh. 1
true positive	0.752	0.944	0.940	0.516
invalid	-	-	0.040	0.484
false positive	0.248	0.056	0.020	0

**Table 6:** Hyperparameters for training PointNet.

Parameter Name	Symbol	Values	Best Value (Model 17)
number of points	*n*	[128, 512, 2048]	2048
batch size	*b*	[32, 128]	128
augmentation factor	*f*	[10, 50, 100]	100

**Table 7:** Accuracies of selected models for best epoch after 800 epochs of training.

Model	Train Time	${\mathrm{acc}}_{\mathrm{train}}$	${\mathrm{acc}}_{\mathrm{val}}$	${\mathrm{acc}}_{\mathrm{test}}$	${\mathrm{acc}}_{\mathrm{test},\mathrm{noise}}$
17	20 h 11 min	0.994	0.970	0.833	0.967
2	5 h 9 min	0.990	0.973	0.600	0.650

## Data Availability

The data used can be obtained from the authors upon reasonable request.
